# Morphological and Mechanical Properties of the Quadriceps Femoris Muscle-Tendon Unit From Adolescence to Adulthood: Effects of Age and Athletic Training

**DOI:** 10.3389/fphys.2019.01082

**Published:** 2019-08-27

**Authors:** Georgios Charcharis, Falk Mersmann, Sebastian Bohm, Adamantios Arampatzis

**Affiliations:** ^1^Department of Training and Movement Sciences, Humboldt-Universität zu Berlin, Berlin, Germany; ^2^Berlin School of Movement Science, Humboldt-Universität zu Berlin, Berlin, Germany

**Keywords:** adolescent athletes, tendon stiffness, muscle strength, muscle architecture, muscle-tendon imbalances

## Abstract

The combined effects of mechanical loading and maturation during adolescence are still not well understood. The purpose of the study was to investigate the development of the quadriceps femoris muscle-tendon unit from early adolescence (EA), late adolescence (LA) to young adulthood (YA), and examine how it is influenced by athletic training in a cross-sectional design. Forty-one male athletes and forty male non-athletes from three different age groups (EA: 12–14 years, *n* = 29; LA: 16–18 years, *n* = 27; and YA: 20–35 years, *n* = 25) participated in the present study. Maximum strength of the knee extensor muscles, architecture of the vastus lateralis (VL) muscle and patellar tendon stiffness were examined using dynamometry, motion capture, electromyography, and ultrasonography. Muscle strength and tendon stiffness significantly increased (*p* < 0.001) from EA to LA without any further alterations (*p* > 0.05) from LA to YA. Athletes compared to non-athletes showed significantly greater (*p* < 0.001) absolute muscle strength (EA: 3.52 ± 0.75 vs. 3.20 ± 0.42 Nm/kg; LA: 4.47 ± 0.61 vs. 3.83 ± 0.56 Nm/kg; and YA: 4.61 ± 0.55 vs. 3.60 ± 0.53), tendon stiffness (EA: 990 ± 317 vs. 814 ± 299 N/mm; LA: 1266 ± 275 vs. 1110 ± 255 N/mm; and YA: 1487 ± 354 vs. 1257 ± 328), and VL thickness (EA: 19.7 ± 3.2 vs. 16.2 ± 3.4 mm; LA: 23.0 ± 4.2 vs. 20.1 ± 3.3 mm; and YA: 25.5 ± 4.2 vs. 23.9 ± 3.9 mm). Athletes were more likely to reach strain magnitudes higher than 9% strain compared to non-athlete controls (EA: 28 vs. 15%; LA: 46 vs. 16%; and YA: 66 vs. 33%) indicating an increased mechanical demand for the tendon. Although the properties of the quadriceps femoris muscle-tendon unit are enhanced by athletic training, their development from early-adolescence to adulthood remain similar in athletes and non-athletes with the major alterations between early and LA. However, both age and athletic training was associated with a higher prevalence of imbalances within the muscle-tendon unit and a resultant increased mechanical demand for the patellar tendon.

## Introduction

Human maturation describes the tempo and timing of the progress toward the mature state during growth (Mirwald et al., 2002). It is well known that during maturation the muscle-tendon unit is subjected to morphological and mechanical alterations (Kanehisa et al., 1995a; O’Brien et al., 2010; Kubo et al., 2014b). Muscle strength is increasing with age in line with body height and mass (Beunen and Malina, 1988; Kanehisa et al., 1995a; Degache et al., 2010), and increases markedly between 13 and 15 years in both sexes (Kanehisa et al., 1995a). Furthermore, Kanehisa et al. (Kanehisa et al., 1995a,b) reported an increase of the muscle anatomical cross-sectional area with age in parallel with muscle strength and, similarly, a pronounced development between age 13 and 15 years in untrained boys. The functional and morphological development of the muscle seems to continue until adulthood (Kubo et al., 2001, 2014b). On the other hand, there is evidence that the muscle strength in athletes increases most between 12 and 13 years in boys (Degache et al., 2010) and, thus, potentially earlier compared to untrained counterparts. Considering the increased secretion of muscle hypertrophy-mediating hormone levels, which occurs at that age (Vingren et al., 2010; Murray and Clayton, 2013) and is promoted by physical activity (Kraemer et al., 1992; Zakas et al., 1994; Tsolakis et al., 2004), it might even be that morphological changes of the muscle contribute to the adaptive response to increased mechanical loading. For instance, mid-adolescent athletes can already feature adult-like muscle morphology with only minor changes of muscle volume thereafter (Mersmann et al., 2014, 2017b) as well as greater muscle pennation angles compared to similar-aged controls (Mersmann et al., 2016). Thus, it seems possible that even early adolescent athletes already show indications of loading-related hypertrophy and muscle remodeling and that there is an interaction of maturation and superimposed loading that influences the temporal development of muscle during adolescence features (in terms of an earlier development) compared to untrained individuals.

Similar to muscles, tendon properties are also affected by the influence of maturation (O’Brien et al., 2009; Kubo et al., 2014b), including its cross-sectional area, Young’s modulus (as a measure of its material properties based on the stress-strain relationship) and stiffness (as a measure of its mechanical resilience based on the force-elongation relationship). Tendon stiffness is a crucial mechanical property because it influences the transmission of the muscle force to the skeleton and depends on its material properties and dimensions (Butler et al., 1978). Patellar tendon stiffness and its determinants cross-sectional area (CSA), rest length and Young’s modulus were reported to increase during maturation from 9 years to adulthood in humans (O’Brien et al., 2009). In accordance with the previous study, Kubo et al. (2014b) and Waugh et al. (2012) reported that Achilles tendon Young’s modulus was lower in children (9–12 years) compared to adults, and junior high school students (13–15 years) had adult-like material properties. The mechanical changes observed from child- to adulthood may partly be mediated by an increase in the structural integrity of the collagenous network (Rudavsky et al., 2017, 2018). During pubertal growth, tendon length increases in a higher rate compared to the CSA, indicating that increments of tendon stiffness are mainly governed by a change of the material properties (Neugebauer and Hawkins, 2012; Waugh et al., 2012). Since tendons adapt to mechanical loading (Bohm et al., 2015), the increase of mass and muscle strength during maturation may increase the stiffness due to increased tendon loading during the daily weight-bearing tasks and the increased muscle force (Waugh et al., 2012). At the end of adolescence, tendon tissue turnover becomes greatly reduced (Heinemeier et al., 2013), yet the plasticity of the tendon is maintained, mainly in terms of loading-induced changes of the material properties (Bohm et al., 2015).

Irrespective of gains in body mass, superimposed mechanical loading by sports activity further can increase tendon stiffness in adolescence (Mersmann et al., 2017c), which suggests that the development of tendon mechanical properties during maturation might be different in athletes compared to adolescents that do not train systematically. Similar to muscle strength, data on the Achilles tendon of untrained adolescents suggest that the maturation-related increases of tendon stiffness are most pronounced early in adolescence (Kubo et al., 2014a; Mogi et al., 2018). Yet a study of our laboratory on adolescent volleyball athletes suggests that – under the twofold stimulus of maturation and training – major changes of tendon CSA and stiffness might occur later in adolescence compared to the muscular development (Mersmann et al., 2017b). Since there is little information considering muscle and tendon development during adolescence, there is still great uncertainty how maturation affects the muscle-tendon unit, especially in interaction with superimposed loading by means of athletic training. The increase of our understanding regarding this interplay might be of particular importance in terms of recent evidence, which lends support to the idea that an imbalanced development of muscle strength and tendon stiffness might increase the risk of overuse tendon injury (see Mersmann et al., 2017a for a review). An adequate strain applied to the tendon is important and necessary for tendon healthiness and adaptability (Bohm et al., 2015; Wiesinger et al., 2015). For example, mechanical tendon loading that introduce low strain values (∼3%) cannot improve tendon properties (Arampatzis et al., 2007a, 2010). However, if a tendon is repeatedly subjected to very high levels of strain, this might induce overload. In a rodent model, Wang et al. (2013) demonstrated that cyclic application of 9% tendon strain acts degenerative on the tissue and weakens its structural integrity. As ultimate tendon strain is irrespective of species (LaCroix et al., 2013) and considering the average levels of maximum *in vivo* tendon strain observed in humans using ultrasound (e.g., Hansen et al., 2006; Couppé et al., 2009; Mersmann et al., 2016, 2018), strain magnitudes higher than 9.0% during maximum isometric contractions might be indicative for imbalances within the muscle-tendon unit, characterized by the tendon stiffness being too low compared to the strength of the associated muscle (Bohm et al., 2019).

The purpose of this research was to investigate the musculotendinous development during adolescence and how it is influenced by athletic training by means of comparing athletes and non-athletes in three different age groups (i.e., early adolescents: 12–14 years, late adolescents: 16–18 years and adults) under the reasonable assumption that these groups would also substantially differ in terms of maturation. We focused on the quadriceps femoris muscle-tendon unit due to its important contribution to movement performance and susceptibility to overuse injury (Zwerver et al., 2011; Simpson et al., 2016; Nikolaidou et al., 2017). We hypothesized to find higher muscle strength, muscle thickness, pennation angle, and tendon stiffness in athletes compared to non-athlete controls in all age groups. Moreover, we expected to find in athletes the major development of tendon stiffness between late adolescence (LA) and adulthood, yet more timely clear increases of muscle strength (Degache et al., 2010; Mersmann et al., 2017b), which may increase the mechanical demand for the tendon.

## Materials and Methods

### Experimental Design

Eighty-one male participants comprised of athletes (*n* = 41) and untrained controls (*n* = 40) in three age groups [EA: early adolescence (*n* = 29), 12–14 years; LA: late adolescence (*n* = 27), 16–18 years; and YA: young adulthood (*n* = 25), 20–35 years] were included in the study (Table 1). The athletes were recruited from the disciplines American football, volleyball, handball, basketball, judo, kick-boxing, fencing, gymnastics, dancing, hockey, vaulting, track and field, acrobatics, decathlon, and trained at least three times per week for at least 75 min per session. Athletes from endurance sports were excluded, because the sport-specific low-intensity loading is unlikely to be a sufficient stimulus to significantly change the mechanical properties of the muscle-tendon unit (Karamanidis and Arampatzis, 2006; Arampatzis et al., 2007b). The sport activity of the untrained adolescent controls was limited to school sports and a maximum of one session of recreational sports per week, while in adults only the latter applied. None of the participants suffered from any orthopedic abnormality or injury at the lower extremities.

**TABLE 1:** Anthropometrical characteristics of the non-athletes and athletes in the three age groups (EA, early adolescence; LA, late adolescence; YA, young adulthood; means ± standard deviation).

	**Non-athletes**			**Athletes**		
	**EA (*n* = 14)**	**LA (*n* = 13)**	**YA (*n* = 13)**	**EA (*n* = 15)**	**LA (*n* = 14)**	**YA (*n* = 12)**
Age [years]	12.8 ± 0.6^b,c^	17.3 ± 0.8^a,c^	29.0 ± 3.6^a,b^	13.0 ± 0.8^b,c^	17.2 ± 0.8^a,c^	26.3 ± 3.0^a,b^
Body height [cm]^∗#^	159.6 ± 11.0^b,c^	175.1 ± 5.3^a^	179.4 ± 9.6^a^	168.6 ± 12.0^b,c^	183.1 ± 8.4^a^	182.1 ± 8.1^a^
Body mass [kg]^∗^	45.4 ± 10.3^b,c^	70.1 ± 15.0^a,c^	80.7 ± 16.5^a,b^	56.2 ± 11.2^b,c^	72.7 ± 10.4^a,c^	79.5 ± 9.1^a,b^
Femur length [cm]^∗#^	38.7 ± 2.2^b,c^	41.0 ± 1.9^a^	40.6 ± 3.8^a^	39.8 ± 4.1^b,c^	43.8 ± 3.8^a^	42.9 ± 2.9^a^

*^#^Statistically significant effect of activity (*p* < 0.05). ^∗^Statistically significant effect of age (*p* < 0.05). ^*a*^Statistically significant difference to EA (*p* < 0.05). ^*b*^Statistically significant difference to LA (*p* < 0.05). ^*c*^Statistically significant difference to YA (*p* < 0.05).*

The study was carried out in accordance with the recommendations of the Ethics Committee of the Humboldt-Universität zu Berlin. All participants (and their respective legal guardians in the adolescent groups) gave written informed consent in accordance with the Declaration of Helsinki. The measurements of muscle strength (i.e., knee extension moments), vastus lateralis (VL) architecture and patellar tendon mechanical properties were carried out on the dominant leg (i.e., leg used for kicking a ball) following a standardized warm-up consisting of 2–3 min ergometer cycling, ten submaximal isometric contractions, and three maximum voluntary isometric contractions (MVC).

### Measurement of Maximum Knee Joint Moment

For the assessment of the muscle strength of the knee extensor muscles, the participants performed isometric MVCs on a dynamometer (Biodex Medical System 3, Shirley, NY, United States) at 65°, 70°, and 75° knee joint angle (i.e., values at rest measured by the dynamometer; 0° = full knee extension). In our earlier work (e.g., Mersmann et al., 2017c), we found that using these resting angles, the participants reach their approximate optimum angle for force generation during the contractions. The trunk angle was set to 85° (neutral full hip extension = 0°) and the hip was fixed to the dynamometer seat using a non-elastic strap.

Since there are differences between the resultant knee joint moment and the moment measured by the dynamometer due to the changes of the knee joint axis relative to the axis of the dynamometer during the MVC induced by soft tissue deformation and dynamometer compliance, we followed the inverse dynamics approach introduced by Arampatzis et al. (2004). Kinematic data were recorded using a Vicon motion capture system (version 1.7.1; Vicon Motion Systems, Oxford, United Kingdom) integrating eight cameras operating at 250 Hz. Six reflective markers were captured, which were fixed on the following positions: lateral and medial malleolus, the most prominent points of the lateral and medial femoral condyles, trochanter major, and lateral aspect of the iliac spine. Passive knee joint moments due to gravity were recorded as a function of knee joint angle in an additional trial. The participants were instructed to relax the muscles of their dominant leg and then the joint was passively rotated at 5°/s through the full range of motion by the dynamometer. Further, we accounted for the contribution of antagonistic muscle activity to the resultant moment by establishing a linear electromyographic (EMG)-activity – knee flexion moment relationship during submaximal isometric contractions (Mademli et al., 2004). For this purpose, we recorded two additional knee flexion trials featuring an EMG-activity that was slightly lower and higher, respectively, compared to the activity registered during the maximum knee extension trials. The EMG activity of the lateral head of the biceps femoris was recorded using two bipolar surface electrodes (Blue Sensor N, Ambu GmbH, Bad Nauheim, Germany) placed over the mid-portion of the muscle belly with an inter-electrode distance of 2 cm after shaving and cleaning the skin to reduce skin impedance. EMG data was captured at 1000 Hz (Myon m320RX; Myon, Baar, Switzerland) and transmitted to the Vicon system via a 16-channel A-D converter.

### Measurement of Vastus Lateralis Muscle Architecture

For the assessment of the VL architecture, ultrasound images were captured at 60° knee joint angle, which has been reported by Herzog et al. (1990) to be the approximate optimum angle of the VL for force production. A 10 cm linear ultrasound probe (7.5 MHz; My Lab60; Esaote, Genova, Italy; probe: linear array (LA923), depth: 7.4 cm, focal point: 0.9 and 1.9, no image filter) was placed over the belly of the inactive muscle in its longitudinal axis at 60% thigh length, which is the assumed location of the maximum anatomical cross-sectional area (Mersmann et al., 2015). The ultrasound images were analyzed offline using a custom written MATLAB interface (version R2012a; MathWorks, Natick, MA, United States). The upper and deeper aponeuroses were defined by setting three reference points along each aponeurosis and a linear least-squares-fit through these points. Subsequently, the visible features of multiple fascicles were marked manually and a reference fascicle was calculated based on the average inclination of the fascicle portions and the distance of the aponeuroses (Marzilger et al., 2017). The pennation angle refers to the angle between the reference fascicle and the deeper aponeurosis. Fascicle length was normalized to femur length (measured from the greater trochanter to the lateral epicondyle, identified by palpation, by means of a measuring tape).

### Mechanical Properties of the Patellar Tendon

To investigate the force-elongation relationship of the patellar tendon, the ultrasound probe (i.e., similar probe and settings as described previous) was fixed by means of a custom-made knee brace overlying the patellar tendon in the sagittal plane. The participants performed 5 isometric ramp contractions, gradually increasing their effort from rest to maximum in ∼5 s and simultaneously the elongation of the tendon was captured by means of the ultrasound at 25 Hz. The resting knee joint angle for the ramp contractions was set according to the MVC trial in which the highest moment was achieved by the respective participant. The knee joint moments were calculated according the same consideration as described above, applying the inverse dynamics approach and correction for antagonistic activity. Tendon force was calculated by dividing the knee extension moment by the tendon moment arm.

The moment arms were predicted using the regression equation reported by Mersmann et al. (2016) based on sex, body height, and mass. Since the moment arm of the patellar tendon is significantly influenced by the knee joint angle, it was adjusted to the respective knee joint angle position based on the polynomial regression equation suggested by Herzog and Read (1993). The ultrasound images were synchronized with the kinematic and analog data using an externally induced voltage peak. Patellar tendon elongation during the contractions was determined by manually tracking the deep insertion of the tendon at the patellar apex and the tibial tuberosity frame-by-frame using a custom-written MATLAB interface. In order to achieve a high reliability (≥0.95), the force-elongation relationship of the 5 trials of each participant was averaged using the highest common force of the single trials as a peak force (Schulze et al., 2012). Tendon stiffness was calculated between 50 and 100% of the peak tendon force based on a linear regression. As stiffness is influenced by the resting length of the tendon (Butler et al., 1978; Arampatzis et al., 2005), we further calculated the normalized tendon stiffness (i.e., the product of stiffness and rest length) that represents the slope of the force-strain curve.

## Statistics

The statistical analysis was conducted in SPSS (version 20.0; IBM, Armonk, NY, United States). A two–way analysis of variance (ANOVA) was performed with the fixed factors activity (i.e., non-athletes, athletes) and age (i.e., EA, LA, and YA) The Shapiro–Wilk Test was performed to verify the normal distribution of the data and Levene’s test to assess the homogeneity of variances. A Bonferroni-corrected *post hoc* analysis was conducted in the case of a significant age effect or interaction of the factors activity and age. The alpha level for all tests was set to 0.05. The effect size *f* for significant observations were calculated in G^∗^Power (Version 3.1.6; HHU, Düsseldorf, Germany; Faul et al., 2007), based on the partial eta squared or means and pooled standard deviation for non-parametrically tested parameters. The subscript *Activity* and *Age* indicates if the effect size refers to differences between athletes and controls or between age groups, respectively. Effect sizes of 0.1 ≤ *f* < 0.25 will be referred to as small, 0.25 ≤ *f* < 0.5 as medium and *f* ≥ 0.5 as large (Cohen, 1988). Using the whole sample, we calculated the Pearson’s *r* for the correlation of tendon force and stiffness. We further predicted tendon stiffness by tendon force using a linear regression model with group-specific y-intercept and slope constants for each age and activity group, respectively, and compared the residuals of the model prediction with a two-way ANOVA to analyze differences in the association of tendon force and stiffness. The model equation was:

$$y_{i}={\text{c}}_{0}+\beta_{0}\,F_{i}+{\text{c}}_{1}\,{\text{g}}_{i}+\beta_{1}\,{\text{g}}_{i}\,F_{i}+{\text{c}}_{2}\,{\text{l}}_{i}+\beta_{2}\,\text{l}\,F_{i}+{\text{c}}_{3}\,{\text{g}}_{i}\,{\text{l}}_{i}+\beta_{3}\,{\text{g}}_{i}\,{\text{l}}_{i}\,F_{i}+{\text{c}}_{4}\,{\text{a}}_{i}+\beta_{4}\,{\text{a}}_{i}\,F_{i}+{\text{c}}_{5}\,{\text{g}}_{i}\,{\text{a}}_{i}+\beta_{5}\,{\text{g}}_{i}\,{\text{a}}_{i}\,F_{i}+\varepsilon_{i}$$

where *i* is index for participant (1,….,81); *g* is the activity-group variable (non-athlete = 0; athlete = 1); *l* is late adolescent age variable (EA = 0; LA = 1; YA = 0); *a* is young adult age variable (EA = 0; LA = 0; YA = 1); *c* are the intercept constant, β are the slope constants; *F* is tendon force ε is the residual.

We further examined the frequency of individuals that reached strain values greater than 9%, since it has been reported that repetitive strains above 9% can induce catabolic tendon matrix damage (Wang et al., 2013). Though the exceedance of the threshold does not necessarily imply injury, it provides a classification if the mechanical demand for the tendon and risk for fatigue is comparatively high.

## Results

Considering the anthropometric data (Table 1), there was a significant effect of age on body mass (*p* < 0.001, *f*_Age_ = 1.04), but no effect of activity group or activity-by-age interaction (*p* > 0.05). *Post hoc* analysis revealed significantly greater body mass with increasing age of the respective group (*p* < 0.05). There was a significant effect of activity group (*p* = 0.003, *f*_Activity_ = 0.36) and age (*p* < 0.001, *f*_Age_ = 0.83) on body height. Athletes were taller compared to non-athlete controls and EA showed significantly smaller height compared to LA and YA (*p* < 0.001), but there were no significant differences between YA and LA (*p* = 1.0). There was a significant main effect of age and activity (*p* = 0.002, *f*_Age_ = 0.43; *p* = 0.007 *f*_Activity_ = 0.32, respectively) but no activity-by-age interaction (*p* = 0.608) on femur length. EA had smaller femur lengths compared to YA and LA (*p* = 0.002 and *p* = 0.028, respectively), but there were no significant differences between YA and LA (*p* = 1.0).

**FIGURE 1:**
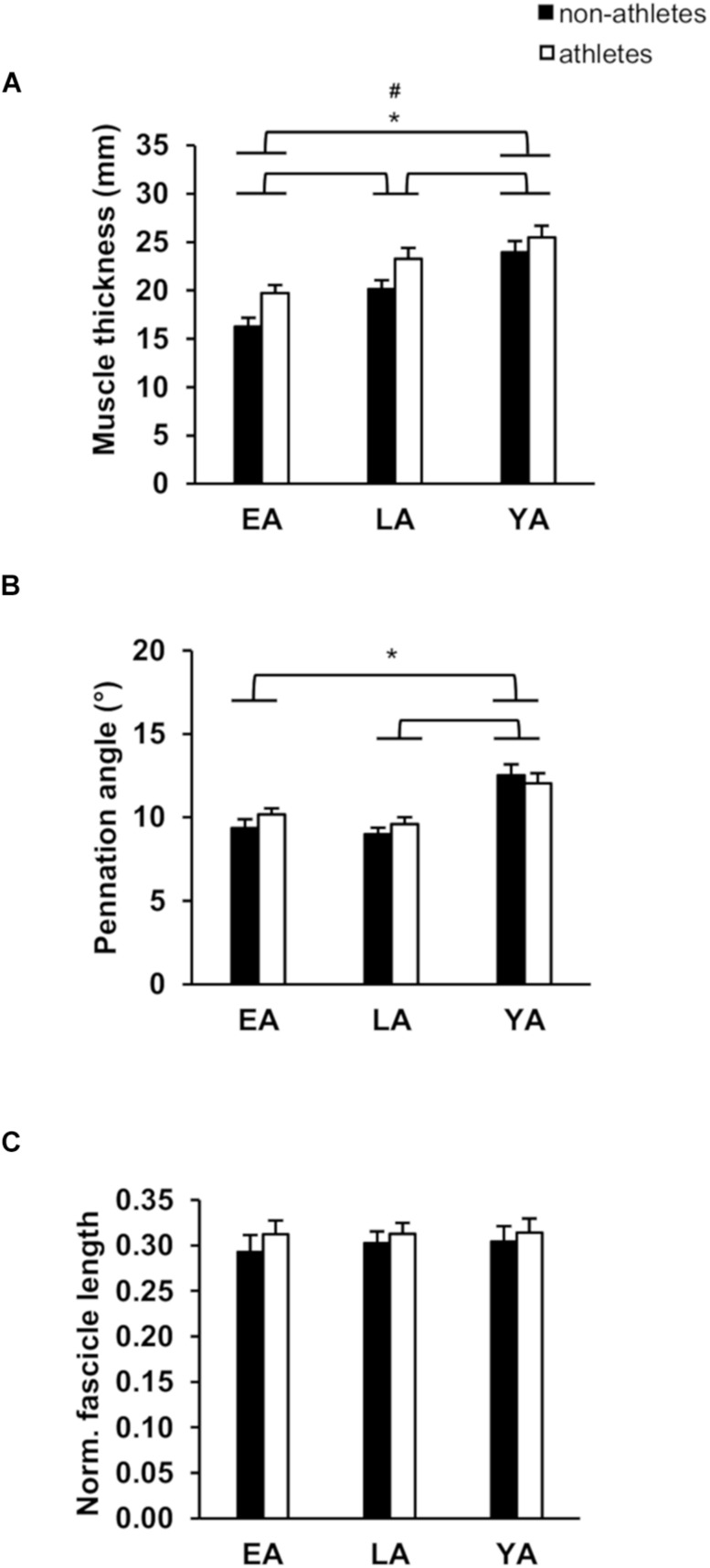
Mean values and standard error (error bars) of vastus lateralis (VL) muscle thickness **(A)**, pennation angle **(B)**, and normalized fascicle length (**C**; normalized to femur length) of non-athletes (black) and athletes (white) in early adolescence (EA), late adolescence (LA), and young adulthood (YA). ^#^Statistically significant effect of activity (*p* < 0.05). ^∗^Statistically significant effect of age (*p* < 0.05).

Considering absolute and normalized muscle strength (normalized to body mass) of the knee extensors, athletes had higher strength compare to non-athletes (*p* < 0.001, *f*_Activity_ = 0.53 for absolute strength and *p* < 0.001, *f*_Activity_ = 0.59 for normalized strength). There was a significant age effect (*p* < 0.001, *f*_Age_ = 1.13 for absolute strength, and *p* < 0.001, *f*_Age_ = 0.64 for normalized strength) but no activity-by-age interaction (*p* = 0.770 and *p* = 0.129 for the absolute and normalized strength, respectively; Table 2). EA had lower absolute strength compared to YA and LA (*p* < 0.001, *f* = 1.14, and *f* = 0.93, respectively) and normalized muscle strength (*p* < 0.001, *f* = 0.51, and *f* = 0.61), but there were no statistically significant differences between YA and LA (*p* = 0.395 and *p* = 1.0). There was no significant effect of age (*p* = 0.743), activity (*p* = 0.370) or activity-by-age interaction (*p* = 0.532 Table 2) on antagonistic co-activation (i.e., antagonistic moment normalized to maximal resultant moment) and tendon resting length (*p* = 0.290, *p* = 0.930, and *p* = 0.505, respectively). We found greater VL muscle thickness in athletes compared to non-athletes (*p* = 0.001, *f*_Activity_ = 0.4) and a significant effect of age (*p* < 0.001, *f*_Age_ = 0.79), but no effect of age-by-activity interaction (*p* = 0.545, Figure 1A). EA and LA had lower (*p* < 0.001, *f* = 0.86, and *p* = 0.001, *f* = 0.48) muscle thickness compared to YA, and EA lower thickness than LA (*p* = 0.007, *f* = 0.41). There was no effect of activity (*p* = 0.473) or age-by-activity interaction (*p* = 0.407) on pennation angle (Figure 1B). However, there was a significant effect of age (*p* < 0.001, *f*_Age_ = 0.6) on pennation angle (Figure 1B). EA, LA both had lower pennation angles compared to YA (*p* < 0.001, *f* = 0.65, and *p* = 0.001, *f* = 0.51), but there were no statistically significant differences between EA and LA (*p* = 0.707). On normalized fascicle length (normalized to femur length), there were no significant effects of age (*p* = 0.903), activity (*p* = 0.299) or age-by-activity interaction (*p* = 0.935; Figure 1C).

**TABLE 2:** Knee joint moments, co-activation (i.e., antagonistic moment normalized to the resultant knee joint moment), tendon resting length, and normalized stiffness of the non-athletes and athletes in the three age groups (EA, early adolescence; LA, late adolescence; YA, young adulthood; means ± standard deviation).

	**Non-athletes**			**Athletes**		
	**EA (*n* = 14)**	**LA (*n* = 13)**	**YA (*n* = 13)**	**EA (*n* = 15)**	**LA (*n* = 14)**	**YA (*n* = 12)**
MVC [Nm]^∗#^	145.2 ± 34.6^b,c^	267.0 ± 72.3^a^	288.2 ± 61.0^a^	202.0 ± 65.7^b,c^	327.3 ± 69.4^a^	367.0 ± 64.7^a^
Normalized MVC [Nm/kg]^∗#^	3.20 ± 0.42^b,c^	3.83 ± 0.56^a^	3.60 ± 0.53^a^	3.52 ± 0.75^b,c^	4.47 ± 0.61^a^	4.61 ± 0.55^a^
Antagonistic co-activation [%]	8.4 ± 4.3	11.1 ± 6.3	8.9 ± 6.1	8.5 ± 6.3	8.1 ± 5.1	8.5 ± 4.5
Tendon resting length [mm]	49.2 ± 8.5	52.0 ± 4.4	51.0 ± 8.4	50.6 ± 6.9	53.0 ± 7.6	48.1 ± 5.9
Tendon normalized stiffness [kN/strain]^∗#^	41.5 ± 11.6^b,c^	57.2 ± 11.1^a^	63.3 ± 15.7^a^	51.0 ± 15.1^b,c^	65.9 ± 14.7^a^	70.5 ± 14.5^a^

*^#^Statistically significant effect of activity (*p* < 0.05). ^∗^Statistically significant effect of age (*p* < 0.05). ^*a*^Statistically significant difference to EA (*p* < 0.05). ^*b*^Statistically significant difference to LA (*p* < 0.05). ^*c*^Statistically significant difference to YA (*p* < 0.05).*

**FIGURE 2:**
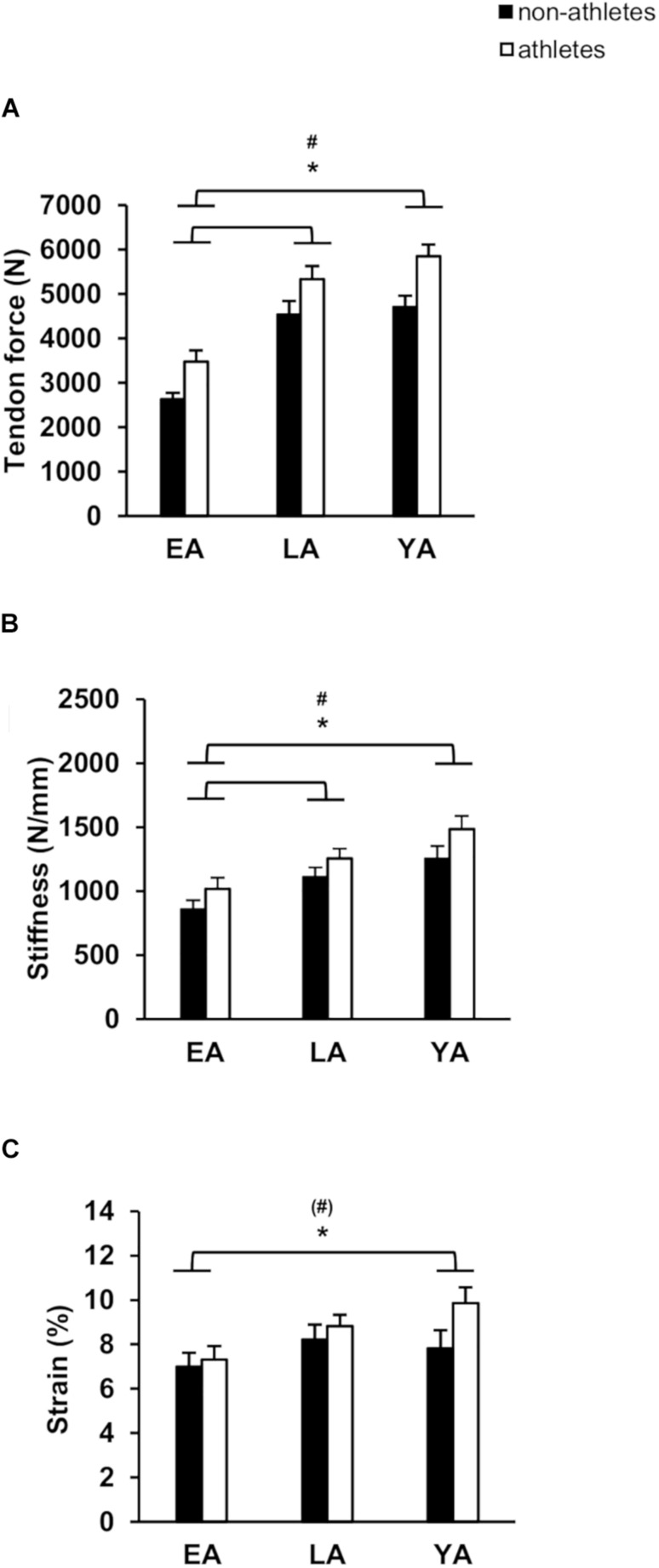
Mean values and standard error (error bars) of patellar tendon: tendon force **(A)**, tendon stiffness **(B)**, and tendon strain **(C)** of non-athletes (black) and athletes (white) in EA, LA, and YA. ^#^Statistically significant effect of activity (*p* < 0.05). ^∗^Statistically significant effect of age (*p* < 0.05). ^(#)^Tendency for an effect of activity, *p* = 0.072.

Patellar tendon maximal force was greater in athletes compared to non-athletes (*p* < 0.001, *f*_Activity_ = 0.52) and there was a significant effect of age (*p* < 0.001, *f*_Age_ = 1.12), but no significant age-by-activity interaction (*p* = 0.772, Figure 2A). EA had significant smaller patellar tendon force compared to LA and YA (*p* < 0.001, *f* = 0.93, and *f* = 1.13, respectively), but there were no significant differences between LA and YA (*p* = 0.602). Athletes had stiffer patellar tendons compared to non-athletes (*p* = 0.013, *f*_Activity_ = 0.31, Figure 2B) and there was a significant effect of age (*p* < 0.001, *f*_Age_ = 0.61). EA had statistically lower patellar tendon stiffness compared to YA (*p* = 0.015, *f* = 0.66) and LA (*p* < 0.001, *f* = 0.42), but there were no significant differences between YA and LA (*p* = 0.104). There was a significant effect of age (*p* < 0.001, *f*_Age_ = 0.66) and a significant effect of activity (*p* = 0.01, *f*_Activity_ = 0.32) on normalized patellar tendon stiffness (Table 2), but no statistically significant activity-by-age interaction (*p* = 0.956). EA had smaller normalized patellar tendon stiffness compared to LA (*p* = 0.001, *f* = 0.55) and YA (*p* < 0.001, *f* = 0.70) but no significant differences between LA and YA (*p* = 0.592). There was a significant effect of age on patellar tendon maximum strain (*p* = 0.028, *f*_Age_ = 0.33; Figure 2C). EA had lower tendon strain compared to YA (*p* = 0.039, *f* = 0.33), but there were no statistically significant differences between EA and LA (*p* = 0.120), or LA and YA (*p* = 1.0). There was a tendency toward an effect of activity on patellar tendon strain (*p* = 0.072, *f*_Activity_ = 0.22), but no age-by-activity interaction (*p* = 0.389).

**FIGURE 3:**
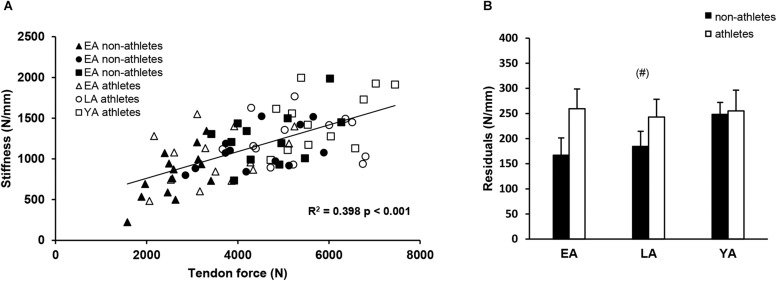
**(A)** Correlation of tendon force and stiffness of non-athletes (black) and athletes (white) in early adolescence (EA, triangles), late adolescence (LA, circles), and young adulthood (YA, squares). **(B)** Means and standard error (error bars) of the residuals of the group-specific linear regression model (see section “Materials and Methods”) of non-athletes and athletes in EA, LA, and YA. ^(#)^Tendency for an effect of activity, *p* = 0.098.

**FIGURE 4:**
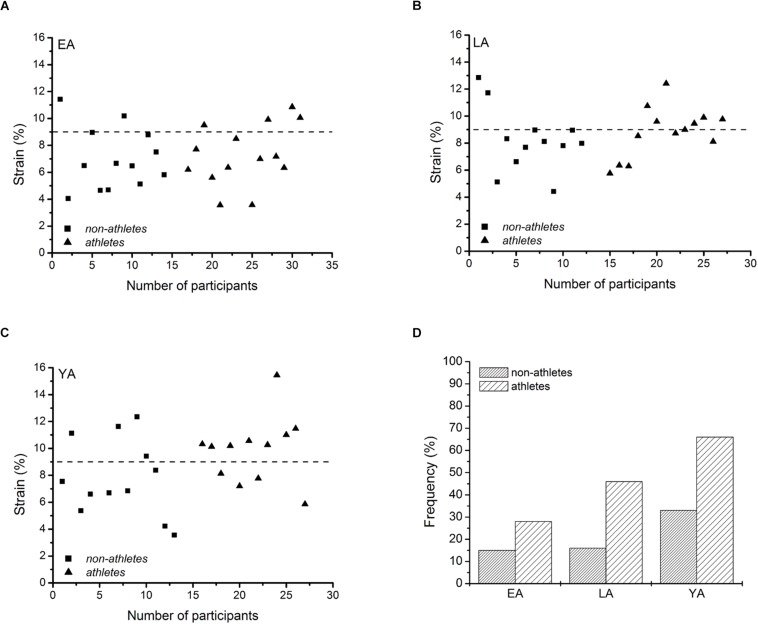
Individual patellar tendon strain values during maximal isometric contractions of non-athletes (squares) and athletes (triangles) in early adolescent (**A**; EA), late adolescent (**B**; LA) and young adulthood (**C**; YA), and frequency of cases with strain values greater than 9% for each group **(D)**.

There was a significant correlation between tendon force and tendon stiffness (*r* = 0.631, *p* < 0.001, Figure 3A) for the whole investigated group of participants. The residuals of the regression model that included group-specific terms showed a tendency for an activity effect (*p* = 0.098) and no effect of age (*p* = 0.524) or age-by-activity interaction (0.536, Figure 3B). Examining the individual tendon strain values during the maximum isometric contractions, it is notable that athletes were more likely to reach strain magnitudes higher than 9% strain compared to non-athlete controls (frequency in athletes: 28–66% and in non-athletes: 15–33%, Figure 4). Further, the frequency of individuals that reach strain values greater than 9% increased from EA to YA in both athletes and non-athletes (Figure 4).

## Discussion

The present cross-sectional study investigated the development of quadriceps femoris muscle strength, VL architecture and patellar tendon mechanical properties during adolescence and how it is influenced by athletic training. The results show that, both muscle and tendon were affected by athletic training, demonstrating greater muscle strength, tendon stiffness and VL thickness in athletes compared to non-athlete controls. However, although the absolute values were different between athletes and controls, the development of muscle strength, tendon stiffness and VL thickness from early adolescent to adulthood did not differ significantly, indicating a similar effect of maturation on muscle-tendon properties in both groups.

There was a marked increase in muscle strength of the knee extensors from early to late adolescents in both athletes (62%) and non-athletes (86%) and no differences between late adolescent and young adults. These findings are in agreement with earlier studies reporting the effect of maturation on the muscle strength development (Kanehisa et al., 1995a, b; Landi et al., 2017). In all investigated age-groups, athletes demonstrated greater muscle strength and VL muscle thickness compared to non-athletes, evidencing a training-induced adaptation in the knee extensor muscles. Furthermore, the increased VL muscle thickness indicates muscle hypertrophy even in the EA as a consequence of intensive athletic training. Similarly, a marked increase from EA to LA without any differences between LA and YA and a clear effect of athletic training was found in patellar tendon stiffness and normalized patellar tendon stiffness. In a previous study (Mersmann et al., 2017c), we reported greater patellar tendon stiffness in late-adolescent Volleyball athletes compared to untrained controls, demonstrating the tendon’s responsiveness to mechanical loading in this age. Our current study provides additional evidence that tendons adapt to increased mechanical loading and enhance their stiffness already in EA. In our EA participants, the average enhancement in patellar tendon stiffness due to training was ∼25%, which can be interpreted as clear and functionally relevant adaptation.

The main alteration in muscle strength and tendon stiffness due to maturation seems to occur between EA and LA. In this stage also the main changes in the femur length, body height and body mass occurred, which indicates an analogous development of the functional and mechanical muscle-tendon properties with the skeletal system. Further, normalized fascicle length (fascicle length/femur length) was similar between all age groups and without any athletic training effect, indicating that during maturation fascicle length development is proportional to bone growth. To our knowledge, this is the first study investigating the interaction between athletic training and age in both muscle and tendon properties during adolescence. We hypothesized an effect of athletic training on the development in muscle and tendon properties during adolescence because the level of the androgenic hormones (e.g., testosterone), which promote protein synthesis and, thus, muscle hypertrophy (Murray and Clayton, 2013; Lundberg, 2017), is different in each stage of maturation and can additionally be affected by athletic training (Kraemer et al., 1992; Zakas et al., 1994; Tsolakis et al., 2004). The absence of any age-by-activity interaction indicates that, irrespective of the marked differences in the average levels of muscle strength and tendon stiffness, the course of the development of these muscle-tendon unit properties with maturation is similar in athletes compared to non-athletes. This is somewhat in contrast to our earlier assumptions (Mersmann et al., 2017a) and the conclusion of earlier meta-analyses (Behringer et al., 2010; Moran et al., 2017) that the trainability of muscle strength and the anabolic response of muscles to mechanical stimuli would increase during adolescent maturation, which we thought would affect the course of muscle-tendon development with increasing differences between the athletes and controls with age. Though the systemic basal levels of sex and growth hormones (Murray and Clayton, 2013) and the endocrine response to exercise increase with maturation and influence muscle and tendon protein metabolism (Rooyackers and Nair, 1997; Hulthén et al., 2001; Doessing et al., 2010; Hansen and Kjaer, 2014), the local responses of the muscle-tendon unit to training seems not to be a simple function of the maturation-related changes of the basal levels and load-induced secretion of systemic hormones. For instance, research that directly compared the effects of training in states of high or low concentrations of circulating endogenous hormones found no differences in the intramuscular anabolic signaling (Spiering et al., 2008), acute protein synthesis (West et al., 2009), or the local functional and morphological response to repeated training sessions (West et al., 2010).

In our study, we found an effect of age on tendon strain during maximum contractions with significantly higher tendon strain in adults compared to EA, indicating a disproportionate increase of tendon force compared to stiffness with increasing age. Further, although statistically not significant, the strain values during the maximum isometric contractions as well as the residuals of the regression model predicting tendon stiffness by tendon force were in tendency greater in athletes (*p* = 0.072 and *p* = 0.098, respectively). When examining the individual strain values reached during the maximum isometric contractions in all investigated age groups, it is notable that it was more likely in athletes that individuals reached strain magnitudes higher than 9%, which is indicative of imbalances within the muscle-tendon unit and resultant high mechanical demand for the tendon. Further, the frequency of strain values over 9% increased from EA to YA independent of activity status. These observations lend support to the idea that both athletic training and maturation can lead to an increased prevalence of imbalances between muscle strength and tendon stiffness. Several studies (Lian et al., 2005; Zwerver et al., 2011; Cassel et al., 2015; Simpson et al., 2016) reported a similar phenomenon for the prevalence of tendinopathy with regard to maturation and athletic training (i.e., increased prevalence from EA to YA and in athletes). An increase of overall tendon strain has been shown to increase local tissue strains at the common site of structural degeneration in patellar tendinopathy (Lavagnino et al., 2008). Further, we recently found an association of tendon strain and its structural integrity in adolescent basketball players as well as increased strain and impaired tendon microstructure in a subgroup with tendinopathy (Mersmann et al., 2019). Thus, imbalances between muscle strength and tendon stiffness developing during maturation and with athletic training, repetitively subjecting the tendon to high levels of strain, might be a risk factor in the etiology of overuse-induced tendinopathy as well as the common background tendinosis or the, rather rare, tendinitis. Further, one might speculate that an increase of tendon strain during muscle contraction might lead to a maltracking of the patellar, redistribution of loads at the patellofemoral contact area and, in consequence, patellofemoral pain (Powers et al., 2017), which is also common in adolescents (Rathleff, 2016). Though the association of musculotendinous imbalances to mechanisms of overuse injury warrants experimental evidence, from a preventive point of view, the integration of a specific training that increases tendon stiffness and facilitates a balanced adaptation between muscle and tendon might be an important approach for the athletic practice. Previous research of our group indicates that an effective training stimulus for tendon adaptation is a combination of high loading magnitude, an appropriate loading duration in every repetition (i.e., 3 s) and repetitive loading (Arampatzis et al., 2007a, 2010; Bohm et al., 2014). In children, the development of resistance training competency should precede the application of high loads (Lloyd et al., 2014), yet it has already been shown that specific tendon training in accordance to the exercise recommendations above can be successfully applied in children to increase their tendon stiffness (Waugh et al., 2014). A more comprehensive discussion of tendon training in children and adolescents for the prevention of muscle-tendon imbalances and tendinopathy and specific exercise recommendations can be found elsewhere (Mersmann et al., 2017c).

A limitation of the present study is the lack of control for biological age. However, the assessment of skeletal age involves exposure to radiation and, in addition to the perceived invasiveness, the accuracy of grading the secondary sex characteristics is rather low (Schlossberger et al., 1992; Taylor et al., 2001; Slough et al., 2013), which is a particular problem for small sample comparisons. Estimations of maturity based on anthropometric data are a tempting alternative, yet these predictions cannot account for the considerable variation in anthropometry at a similar stage of maturity. As we included athletes from sports in which body height is a selection criterion (e.g., basketball and volleyball) and, as a result, our athletes were significantly taller compared to the non-athlete controls, any anthropometry-based prediction would also suggest a higher level of maturity in athletes. Even if that might not reflect actual differences in biological age, we cannot rule out differences in maturity. While maturity-related differences in physical characteristics have been reported to be largely eliminated in non-athletes and athletes aged 16–18 (Malina et al., 2004, 2013), the differences observed between athletes, and non-athletes need to be interpreted with care considering the EA group. On the other hand, it seems very unlikely that the clear differences in calendric age between age-groups would not be representative for different stages of maturity. Therefore, we do not believe that our conclusions considering the effects of maturation are affected by the lack of an assessment of actual maturity. Finally, due to the inherent limitations of cross-sectional studies, further longitudinal research is needed to confirm the development of the musculotendinous system and its interaction with mechanical loading indicated by our data.

## Conclusion

In conclusion, the present study provides evidence that aside from higher levels of muscle strength, muscle thickness and tendon stiffness in athletes, the development of the properties of the knee extensor muscle-tendon unit from early-adolescence to adulthood is similar in athletes and non-athlete controls, with the major alterations occurring between early and LA. The frequency of imbalances in the quadriceps femoris muscle-tendon unit seem to increase with both age and athletic training during the adolescence-to-adulthood development and result in an increased mechanical demand for the patellar tendon. Therefore, we recommend to introduce specific intervention protocols in the athletic training practice in order to support a balanced adaptation between muscle and tendon.

## Ethics Statement

This study was carried out in accordance with the recommendations of the Ethics Committee of the Humboldt-Universität zu Berlin. All participants (and their respective legal guardians in the adolescent groups) gave written informed consent in accordance with the Declaration of Helsinki.

## Author Contributions

GC and AA conceived the experiments. GC, FM, and SB performed the experiments. GC analyzed the data. FM, SB, and AA substantially contributed to the data analysis. GC, FM, and AA interpreted the data and drafted the manuscript. SB made important intellectual contributions during revision. All authors approved the final version of the manuscript and agreed to be accountable for the content of the work.

## Conflict of Interest Statement

The authors declare that the research was conducted in the absence of any commercial or financial relationships that could be construed as a potential conflict of interest.
